# Supplementary material for: Are carbon emissions trading and green financial instruments synergistic? -Comprehensive quantitative research based on content analysis
Source: PLoS One. 2024 Mar 7;19(3):e0298601. doi: 10.1371/journal.pone.0298601 (PMC10919601; doi:10.1371/journal.pone.0298601)
Supplement: S1 Data — (ZIP) [file pone.0298601.s001.zip › Data/地级市Z_F工作报告.zip!/╜¡╬≈/╛┼╜¡╩╨/└┤╫╘╣1⁄2╓┌║┼í╛┬φ┐╦╩2╛▌═°í┐_2017.docx]

各位代表：

　　现在，我代表市人民政府，向大会报告工作，请予审议，并请各位市政协委员和列席会议的同志提出意见。

　　一、2016年工作回顾

　　过去的一年，在省委、省政府和市委的正确领导下，面对错综复杂的国内外经济形势，全市上下团结奋进，攻坚克难，较好完成了年初确定的目标任务，实现了“十三五”良好开局。

　　过去一年，我们始终坚持发展为先，打出稳增长组合拳，综合实力不断增强。完成生产总值2096.1亿元，同比增长9.4%；财政总收入415.2亿元，同口径增长12%；8个县（市、区）财政收入过20亿元，九江经济技术开发区突破60亿元；固定资产投资2428.6亿元，增长14.6%；社会消费品零售总额656.8亿元，增长13%；城镇居民和农村居民人均可支配收入30011元、12157元，分别增长8.6%、9.1%；实际利用外资18亿美元，增长10.6%；居民消费价格总水平上涨2.1%；金融机构本外币各项存款余额2610亿元，增长19.5%；各项贷款余额1758亿元，增长20.4%。主要经济指标总量、增幅继续位居全省前列。

　　过去一年，我们始终坚持新工业引领，着力推进转型升级，产业发展不断加快。三次产业比由7.4︰53.3︰39.3调整为7.3︰51.9︰40.8。“新工业十年行动”开局良好，实现规模以上工业企业主营业务收入5487.5亿元，增长9.2%；工业增加值1115.7亿元，增长9.3%，其中五大战略性新兴产业增加值425.7亿元，占比提高2.8个百分点；利税666.4亿元，增长16.5%；规上工业企业净增171户；工业用电量115.3亿千瓦时。完成工业固投1863.2亿元，增长20.7%，集中开工工业项目387个。神华电厂、心连心、兄弟制药、泰盛纸业、日昌升、天瑞丰收、中科鑫星新材料等88个亿元以上项目加快建设。九江石化税收首次突破100亿元，成为全省最大纳税企业。九江钢厂利润实现翻番，税收净增2亿元。6个县（市）工业园完成调区扩区，全市园区面积扩大到146.7平方公里。“两化”融合试点企业超过100户。粮食总产量实现“十三连丰”。市级农村综合产权交易中心建成运行。流转土地面积151.9万亩。新增茶叶种植面积3万亩、油茶7.9万亩，新增新型农业经营主体1878家、农产品“三品一标”47个，创建省级农业示范园区9个。修水获评“全国十大魅力茶乡”和“全国茶产业发展示范县”。市文旅集团组建成立。旅游主要指标居全省前列。庐山西海获批省级旅游度假区，武宁入选首批国家全域旅游示范区。实施亿元以上商贸物流项目72个，新增物流企业187家，总数超过1500家。港口货物吞吐量1.1亿吨，增长9%；集装箱吞吐量27.4万标箱，增长7%。九江-重庆国际集装箱直达航线开通运营。获批国家物流标准化试点城市。引进兴业银行、赣州银行、华融证券、平安养老等金融机构入驻；新增“新三板”挂牌企业5家。完成电子商务交易额385亿元，增长51%；庐山市获评全国电子商务进农村综合示范县（市）。总部经济、楼宇经济、文化创意、工业设计、中介服务等新兴服务业蓬勃发展，服务业增加值占GDP比重突破40%。

　　过去一年，我们始终坚持改革开放创新，破解制约发展难题，发展活力不断释放。坚持以供给侧结构性改革为主线，集中抓好“三去一降一补”。提前完成去产能年度任务，商品房去化时间减少10.2个月。制定“1+6+X”政策措施，大力开展降成本、优环境专项行动，出台优惠政策29条，为企业降本增效103.7亿元。发放“财园信贷通”102.6亿元，总量、增幅连续三年全省第一。“营改增”全面推开，“五证合一”“一照一码”登记制度启动实施，新登记注册企业1.4万户，增长35.9%；新增个体工商户3.7万户，增长29%。农村土地“三权分置”、集体产权制度、农业保险、国有林场等改革加快推进。设立庐山市、濂溪区，庐山管理体制改革取得历史性突破。共青城市和永修县部分区域纳入赣江新区。获批国家级产城融合示范区。破解了一批土地出让、城市建设、国企改革等历史遗留难题。组建市储备粮油公司，完成九江长运公司并购重组，清理“僵尸企业”80多家。九江港进口肉类指定口岸获批筹建，进口粮食指定口岸运营良好。出台推动创业创新政策30条。成立九江产业技术研究院。完善政、校、企科技成果对接转化机制，转化科技成果65项。新增博士后创新基地2家、国家级科技企业孵化器1家，恒盛科技（创业）园跻身全国50强。新增高新技术企业26家。新增发明专利申请量416件，增长72.6%。新增中国驰名商标2件。湖口县、共青城市获批省级高新区。

　　过去一年，我们始终坚持统筹城乡发展，加强基础设施建设，支撑平台不断夯实。全市城镇化率52.2%。完成《九江都市区总体规划》纲要编制和八里湖中央商务区城市设计，启动《九江城市总体规划》修编，出台“智慧九江”总体规划纲要。中心城区实施重点城建项目208个，完成投资88亿元。新区的通岭大道、兴城南大道立交、八里湖环湖绿道、九龙山森林公园等项目建设完成；园艺三期安置小区、中央商务区支路、八赛路等项目加快建设。老城改造深入实施，整治边街小巷55条，改造道路9.4万平方米，修复人行道3万平方米，疏通排水管网26公里；改造公交候车亭96座、农贸市场11家，建立惠民蔬菜直销点10个。城市管理“提升年”活动扎实推进，路面、立面、街面整治力度持续加大，市容环境明显改观。县城功能不断完善，共实施城建项目487个，完成投资190亿元。新增高速公路通车里程123公里，新建改造干线公路141.5公里、农村公路1300公里。修平高速、昌九大道（一期）等建成通车，沙阎路完成路面改造，都九高速路基工程基本完工，昌九高速“四改八”全线开工。武九客专基本建成，九景衢铁路加快推进，合安九高铁开工建设。新建、续建码头3个、泊位6个，红光综合枢纽港项目加快推进。完成电网投资16.2亿元，蔡岭、红光和赛城湖等输变电项目建成使用。“光纤入户”实现行政村全覆盖。完成水利投资30亿元，圩堤加固、水库除险、小农水重点县等一批工程建设完成，修复水毁工程1515座（处），建成高标准农田13.2万亩。

　　过去一年，我们始终坚持绿色发展，加大环境保护力度，生态优势不断提升。生态文明试验区建设有序推进，成功创建国家森林城市，县（市）创建省级森林城市实现全覆盖，修水县列入国家重点生态功能区。大力实施“清河行动”，开展了采砂管理专项整治，长江江西段和柘林湖水资源保护成效良好，城市集中式饮用水源地水质达标率保持100%。工业绿色发展三年行动计划启动实施，扎实开展“零点”、取缔“十小”等专项行动，淘汰关闭违法违规建设项目237个，完成主要污染物减排任务。造林绿化23.3万亩，新增省级生态乡镇9个、生态村5个。全面推行“河长制”，建立了水资源“三条红线”控制指标体系，开展了领导干部自然资源资产离任审计试点工作。

　　过去一年，我们始终坚持保障改善民生，加快发展社会事业，民生福祉不断增进。面对1998年以来最为严峻的大洪久汛，全市上下发扬伟大的抗洪精神，万众一心、众志成城，夺取了抗洪抢险救灾的全面胜利，确保了人民群众的生命安全和江河湖库堤坝安全，得到中央领导和省委、省政府充分肯定。全年财政民生支出359.5亿元，占总支出77.2%。脱贫攻坚首战告捷，9.12万人脱贫摘帽，117个贫困村退出。全市完成棚户区改造拆迁2.9万户，其中中心城区1.02万户；改造农村危房1.03万户。新增城镇就业6.5万人，购买公益性岗位7088个。企业退休人员养老金每人每月增加170.8元。在全省率先建立困难老人护理补贴制度，惠及5000多人。建成“放心粮油”供应网点223家。财政教育投入82.9亿元，新建改造校舍面积113万平方米。完成学前教育第二期三年行动计划，新建公办幼儿园53所。9个县（市、区）通过义务教育发展均衡县国家认定。共青科教城两所院校建成开学。发放教育资助金2亿多元，惠及学生近13万人。城乡居民医保参保率95%以上，人均筹资标准提高到570元。低保、五保提标惠及27.3万人，大病医疗救助17.6万人次。积极创建国家公共文化服务体系示范区，寻庐文化惠民工程通过国家验收。举办市第十二届运动会。城市公立医院综合改革全面启动，分级诊疗制度建设稳步推进，“全面两孩”政策稳妥实施。全国第三次农业普查工作顺利开展，“七五”普法全面启动。严格落实包案责任，化解了一批信访积案和信访问题。食品药品安全形势持续好转，安全生产形势保持稳定。严厉打击违法犯罪，“平安九江”“法治九江”“诚信九江”建设扎实推进，社会大局和谐稳定。

　　审计、气象、水文、盐业、通信、无线电管理、档案、司法、国安、保密、编制、地方志、对台、外事侨务、民族宗教、驻外联络等工作得到新加强；国防动员、民兵预备役、双拥创建、人民防空、防震减灾、消防等工作取得新成绩；妇女儿童、老龄、残疾人、志愿者、红十字和慈善等事业实现新发展。

　　过去一年，我们始终坚持打造实干政府，着力优化发展环境，自身建设不断加强。扎实开展“两学一做”学习教育，认真落实中央“八项规定”精神和国务院“约法三章”要求，坚持把纪律挺在前面，大力整治“四风”。坚持依法行政，建立了市政府常务会学法、规范性文件合法性审查和重大行政执法行为报备制度。自觉接受人大的法律监督和政协的民主监督，人大代表建议和政协委员提案办结率100%。电子政务和网上办事加快推广。“民声直通车”办理意见、建议、诉求8607件，办结率96.4%。深入推进“放管服”改革，网上审批系统上线运行，精简行政审批事项248项。取消调整行政审批中介服务事项81项，清理规范市本级公共服务事项774项。深入推进“违插”治理长效化，大力开展侵害群众利益专项整治行动。“三公”经费网上监管平台实现全覆盖。

　　各位代表，回顾一年来的政府工作，我们深切感受到：所有成绩的取得，得益于省委、省政府和市委的坚强领导，得益于市四套班子的共识共为，得益于全市人民的团结奋进。在此，我代表市人民政府，向各位人大代表、政协委员，向各行各业的广大干部群众，向离退休老同志，向各民主党派、工商联、无党派人士、各人民团体，向驻浔人民解放军和武警官兵，向中央和省驻浔单位，向所有关心、支持和参与九江建设的各界朋友们，表示衷心的感谢并致以崇高的敬意！

　　需向代表说明的是，受经济下行压力较大等因素影响，GDP、固定资产投资、居民收入低于预期目标。我们清醒地看到，当前发展中还存在不少困难和问题，主要表现在：经济总量不大、发展不足，加快发展的任务仍然艰巨；产业结构不优，创新能力不强，转型发展的任务仍然艰巨；人才、资金、土地等要素趋紧，环境承载压力加大，绿色发展的任务仍然艰巨；县域经济实力不强，城镇化水平不高，统筹发展的任务仍然艰巨；部分群众生活还比较困难，影响和谐稳定的因素依然存在，共享发展的任务仍然艰巨；发展环境有待进一步优化，依法行政水平有待进一步提高，干部作风有待进一步加强。对此，我们将高度重视，采取更加有力的措施，努力予以解决。

　　二、2017年工作安排

　　2017年是党的十九大召开的喜庆之年，是供给侧结构性改革的深化之年，也是全面落实市第十一次党代会决策部署的关键之年。尽管国内外形势错综复杂，经济下行压力持续加大，但随着长江经济带、生态文明试验区、赣江新区等重大国家战略的深入实施，多年来积蓄能量加速释放，独特优势将进一步放大，希望大于困难、机遇大于挑战，九江正处于大有作为的“黄金期”和跨越发展的“窗口期”。我们必须科学应对面临的挑战，抓住用好难得的机遇，坚定信心，顺势而为，乘势而上。

　　今年政府工作的总体要求是：以党的十八大、十八届三中、四中、五中、六中全会精神为指导，深入贯彻习近平总书记系列重要讲话精神和治国理政新理念新思想新战略，按照中央经济工作会、省十四次党代会、省经济工作会、市十一次党代会的部署要求，以“新工业十年行动”带动“五化”协同，以聚力创新开放加快绿色发展，着力实施“重大项目落实年”，兴实体经济，扩有效投入，添发展动能，促民生改善，强实干作风，打好脱贫攻坚战，推动经济平稳健康较快发展和社会和谐稳定，奋力迈出九江全面崛起、百姓更加幸福新步伐，以优异成绩迎接党的十九大胜利召开。

　　按照市委“在‘稳中求进’的基础上力求‘稳中快进’，在‘稳中快进’的进程中体现‘既新又好’”的总要求，全市经济社会发展主要预期目标是：生产总值增长9.5%左右；财政总收入增长7%左右；固定资产投资增长15%以上；社会消费品零售总额增长12.5%左右；外贸出口增长1%左右；实际利用外资增长10%以上；规模以上工业增加值增长9.5%左右；城镇居民和农村居民人均可支配收入分别增长9.5%左右和10%左右；价格总水平涨幅控制在3%左右；完成省政府下达的节能减排任务。

　　为实现上述目标，重点抓好十项工作：

　　（一）聚焦做大总量、提升质量，促进产业集聚集群集约，开辟新工业发展的新天地。坚持把新工业作为振兴实体经济的重中之重，持续推进“众创业、个升企、企入规、规转股、扶上市、育龙头、聚集群”，促进工业发展转型升级、量质齐升。确保实现主营业务收入5900亿元、工业固投2000亿元。

　　做大做强主导产业。坚持把“新工业十年行动”作为加快工业发展的主引擎，做大产业规模，促进产业集聚，着力构建九江特色的工业产业体系。健全完善产业推进“六个一”机制，严格落实新工业发展目标考核奖励办法。加快打造五大千亿产业，力争实现主营业务收入4400亿元，其中现代轻纺产业主营业务收入1200亿元，装备制造产业突破1000亿元，石油化工产业820亿元，钢铁有色产业800亿元，电力能源产业500亿元。培育壮大战略性新兴产业，以新材料、新能源、电子电器、生物医药、绿色食品等产业为重点，引进、培育一批产业龙头，加快形成产业集群，力争战略性新兴产业实现增加值450亿元，占比提高2-3个百分点。

　　大力培植骨干龙头。坚持把骨干企业作为带动产业集聚的主力军，通过兼并重组、股改上市、裂变扩张，形成一批有带动作用的产业新龙头。依托现有骨干企业，推动产业链向两端延伸，带动配套企业发展，打造全链式产业集群。加快推进企业技改扩能，实施重大工业技改项目100个，重点抓好九江石化炼油结构调整、星火有机硅50万吨、赛得利化纤50万吨、巨石玻纤30万吨等扩能项目达产达标；加快企业信息化改造，重点培育“两化”融合试点企业100户。通过靠大联强、扩能升级，推动中小企业做大、优势企业做强。力争新增规模以上企业100户。

　　推动园区转型升级。坚持把园区作为打造产业集群的主阵地，提高园区产业集聚度和投入产出率，推动园区转型升级、提质发展。力争修水、武宁工业园区主营业务收入过300亿元，瑞昌、永修、德安、共青过400亿元，湖口、濂溪过500亿元，九江经开区过1100亿元。突出主业强园、特色兴园，重点打造石油化工、新材料、装配式建筑、装备制造、化纤、环保石材、木业家居等特色产业园。加快完善园区生产配套、生活服务功能，推进标准化厂房建设，促进产城深度融合，提升园区生态化建设水平，增强园区承载能力。支持共青创建国家级高新区、德安申报省级高新区，支持各地工业园调区扩区。

　　（二）聚焦项目驱动、投资拉动，狠抓重大项目落实，掀起项目建设的新热潮。着力实施“重大项目落实年”，坚持以项目扩投入、稳增长、调结构、促转型，力争完成固定资产投资2800亿元。

　　抓好项目谋划。抢抓国家重大战略机遇，谋划一批打基础、管长远的重大项目。依托主导产业、骨干企业，加快延链补链，谋划一批产业配套项目。围绕夯实发展平台，提升承载能力，增强发展后劲，谋划一批基础设施项目。立足补短板、完善公共服务，谋划一批现代服务业和民生项目。发挥民间投资的主力军作用，谋划总投资不少于100亿元的PPP项目。

　　加快项目建设。重点推进神华电厂、心连心、泰盛纸业、理文盐氟化工、兄弟制药、天瑞丰收、北汽新能源汽车、赛得利二期、日昌升、万润兴业等重大产业项目竣工投产。推进九景衢铁路、合安九高铁、昌九高速“四改八”、都九高速等重大交通项目建设。推进500千伏、220千伏输变电等重大能源项目建设。

　　健全推进机制。建立项目台帐，明确项目领导、牵头部门、责任单位，层层压实责任。加强项目调度，严格“五环链接”抓落实机制，及时协调解决问题。强化项目督查，建立重大项目“督查长”制，成立专门督查办公室，全程跟踪督查。健全项目考核机制，树立“项目为零，实绩为零”导向，以项目论英雄，以项目评能力，以项目定奖惩。

　　（三）聚焦功能完善、人气提升，加强规划建设管理，迈出城市建设的新步伐。坚持“新老联建、八赛联接、九沙联体”，加大旧城改造力度，加快完善新区功能，着力打造世界知名的山水文化名城。

　　优化空间布局。健全城镇规划体系，合理规划都市区、中心城区、卫星城镇，统筹市区与周边县（市）一体化发展。加快《九江城市总体规划》修编，全面完成中心城区各片区控规修编，做好历史文化名城保护等专项规划修编。启动赛城湖组团、高铁新区控规设计。完成九江县撤县设区。

　　完善城市功能。抢抓棚改政策“窗口期”，按照“多拆少建，分拆整建，只拆不建”的原则，科学规划、综合施策、整体推进，中心城区完成棚改拆迁2.6万户。推进庾亮南路等历史文化街区建设。加快实施动高联运、九江火车站站外广场改造、九江高铁枢纽、高速出入口“拆四建二”、庐山机场改造等项目。建成赛城湖大桥，启动寻阳古城、环赛城湖景观工程等项目建设。加快八里湖中央商务区、金融先导区建设，完善周边路网配套和公共服务设施。实施中央商务区综合管廊试点项目，加快给排水等地下管网改造，建设“海绵城市”。

　　提升城市形象。加快实施城市绿化、亮化、净化、美化和城市“双修”工程。实施甘棠湖、南门湖周边建筑和滨江大道亮化提升工程。加强长江排污口整治，推进白水湖、芳兰湖污水处理厂等项目建设。完善提升数字化城管平台，深入开展城市管理“打违治乱”专项行动，扎实推进“做文明九江人”系列活动，认真实施城区烟花爆竹燃放管理条例，不断提升城市管理水平和文明程度。

　　打造特色城镇。加快县城建设，老城区以棚户区改造为契机，加大边街小巷、环境卫生、交通秩序等整治力度，进一步改善人居环境；新城区重点完善市政配套和服务功能，加快集聚人气，提升产业承载能力。加快推进重点集镇建设，明确产业定位、完善服务功能、突出建筑特色，打造一批现代制造、商贸物流、休闲旅游、山水田园、历史文化等特色小镇。启动海会云雾小镇建设，加快牯岭艺术小镇建设，抓好艾城国家新型城镇化综合试点工作。

　　（四）聚焦发展提速、比重提升，繁荣发展第三产业，打造转型发展的新引擎。突出生产与生活并重、现代与传统并举，补齐服务业发展短板，增强经济增长拉动力。

　　壮大文化旅游。做好旅游招商，推动“环庐山、大庐山、泛庐山”旅游创意项目落地。建成庐山索道，完成庐山南门周边环境提升，加快推进长江风光带、庐山西海体育四大中心、环西海旅游公路等项目建设。支持庐山西海创建国家5A级景区，庐山市和庐山西海创建国家级旅游度假区。办好全省旅游产业发展大会。发挥文旅集团作用，引进实力强、善经营的投资主体，提高旅游项目档次。建设一批乡村旅游点，推动乡村旅游提档升级。进一步规范行业管理，整顿旅游市场秩序，提高旅游服务水平。全年接待游客总人数、旅游总收入增长15%以上。

　　做强金融产业。加大金融机构和高端人才引进力度，集聚金融要素，完善金融市场体系。加快运作“1+6”产业投资引导基金。按照现代企业制度改造政府性投融资平台，打造全省一流的国有资本运营公司。盘活政府资源资产，把资源变资产，资产变资本，资本变股本。加大信贷投放力度。扩大直接融资规模，全力推动九江银行上市，力争新增10家“新三板”挂牌企业。用足国家金融扶贫政策，支持修水县引进、培植一批企业申报上市。

　　繁荣商贸物流。扎实开展全国物流标准化试点工作，促进电商和物流业协同发展。组建、引进一批物流集团，新建改造一批示范物流园。做大联盛易佳、永修云硅、万其、颐高等电商平台，打造特色品牌。推进电子商务进农村、进社区，加快发展跨境电商，创立跨境电商产业园。支持共青城创建国家电子商务示范基地。

　　（五）聚焦改革攻坚、扩大开放，打造双向开放高地，拓展经济社会发展的新空间。向改革要动力，向开放要活力，更大力度推进高水平双向开放。

　　加快改革攻坚。深化供给侧结构性改革，抓好“三去一降一补”任务落实。积极稳妥去产能，严格执行环保、能耗等法律法规和标准，加快传统产业改造升级，坚决完成省下达的去产能任务；因地制宜去库存，提高货币化安置率，加强住房市场监管，促进房地产市场平稳健康发展。持续抓好降成本、优环境政策措施落地，不断降低企业成本。推进农垦企业改革，做好国有企业改革扫尾工作，处理好改革历史遗留问题。深化“放管服”、国资、财税金融等领域改革，抓好九江经开区、庐山西海、八里湖新区等管理体制改革。

　　全力招大引强。继续把招商引资作为经济工作的“头号工程”。坚持引资、引技、引智并举，围绕重点产业、龙头企业，运用以商招商、产业招商、科技招商、资本招商、环境招商、专业招商等方式，着力引进一批科技含量高、带动能力强的大企业、大集团。精心筹划准备，积极沟通对接，提高“央企入赣”“长江经济带（九江）新工业新经济投洽会”“赣台会”等活动实效。全力推进外贸稳增长调结构，加快发展加工贸易，提升外贸企业内生动力。支持企业“走出去”，拓展国际发展空间。

　　完善开放平台。深入推进昌九一体化，支持永修、共青积极参与赣江新区建设，发挥新区平台政策优势，在引进产业和项目上取得重大突破。加速进境肉类指定口岸建设，申报原木、整车、水果、冰鲜水产品等指定口岸。加快电子口岸建设，推动九江港开放升级。做好综合保税区申报工作，加快共青城中俄国际商贸城建设。整合岸线资源，推进红光综合枢纽港、赤湖公用码头建设，建成彭泽、瑞昌、湖口作业区疏港通道。大力发展临港经济，把九江打造成为“服务全省、辐射周边”的航运中心。积极参与沿江经济协作区建设，大力承接发达地区先进制造业和先进技术转移。

　　优化营商环境。加大企业帮扶力度，落实好“领导挂点企业”“百个部门帮百企”等帮扶机制，继续开展政企“茶叙会”，帮助企业破解发展难题。充分发挥企业投诉中心、“工业110”服务功能，健全投诉快速查处机制。积极开展“企业评部门”活动，努力营造亲商、安商、富商的良好环境。

　　（六）聚焦增效增收、搞活放活，推进农业供给侧改革，构建城乡协调发展的新格局。按照“稳粮、优供、增效”的要求，增加绿色优质农产品供给，改善农村生产生活条件，不断夯实农业农村发展基础。

　　加快发展现代农业。落实粮食安全责任制，稳定粮食综合生产能力。建设高标准农田8万亩。大力发展“两茶一水”等特色产业，新增虾蟹养殖面积3万亩、茶叶2万亩、油茶4万亩。按照“一县一园”要求，引导资金、科技、人才等向农业园区集聚，高标准建设一批现代农业示范园，支持永修创建国家现代农业示范园区。培育发展村集体经济，开展多种形式的“村庄经营”，实现资源变资产、资金变股金、农民变股东，增强村集体经济发展实力和活力。支持农业龙头企业做大做强，新增龙头企业20家以上。

　　着力建设美丽乡村。围绕建设富裕美丽新农村，突出村庄整治、环境美化、乡风文明、和谐平安四大重点，加快推进农村基础设施建设，整治一批自然村组，改善农村人居环境。抓好农村生活垃圾和污水处理，全面整治农业面源污染。推进水利工程建设和水毁工程修复，治理水土流失面积74平方公里。新建农村公路1200公里。强化农民建房管理，防止超高超大、乱搭乱建。树立文明乡风，形成良好社会风尚。

　　创新农业农村发展体制机制。推进农村土地“三权分置”改革，促进土地有序流转，发展多种形式适度规模经营。完善农村产权交易平台，加强农村集体“三资”网络监管。抓好小型水利工程管理体制改革。创新金融支农方式，积极开展林权抵押贷款业务。

　　（七）聚焦大众创业、万众创新，夯实创新平台，激发经济发展的新动能。深入实施创新驱动战略，大力发展新经济，加快培育新动能，推动产业迈向中高端。

　　突出创新引领作用。深入推进创新驱动“5511”工程，加强创新成果同产业对接，创新项目同现实生产力对接，实施一批重大科技专项，以技术突破引领产业升级。突出企业创新主体地位，培育一批创新型引领企业和科技型中小企业。新增高新技术企业30家以上。加强知识产权保护和运用，加大专利申请和专利产业化扶持力度，全年专利申请量增长35%以上。

　　强化创新要素保障。整合现有创新平台资源，提升一批双创孵化器，培育一批双创示范基地，搭建一批双创服务平台，大力发展新型众创空间。规划启动鄱阳湖生态科技城建设，支持共青城创建全国双创示范基地，推进湖口高新技术产业化基地建设，力争每个县（市、区）都建有一个科技园区。充分发挥科技创新发展基金作用，引导企业和社会资本加大科技投入，促进科技型企业成长壮大。深入实施人才“尊优计划”和“双百双千”工程，鼓励“浔才回家”，充分发挥人才在创新创业中的决定性作用。

　　营造创新创业氛围。大力推进全民创业，放宽准入门槛，激活民间资本，激发创新活力，增强内生动力。实施高校毕业生就业创业促进计划，支持共青城举办创新创业大赛。完善激励人才、激发创新的体制机制，大力发展新技术、新产业、新业态、新模式、新服务等新经济，让能创新、愿创新的人有机会、有舞台。

　　（八）聚焦生态优先、绿色发展，全力保护生态环境，提升绿色发展的新优势。扎实推进国家生态文明试验区建设，引环保项目，建生态园区，促绿色发展。

　　发展绿色产业。推进项目清洁化，严格落实项目节能评估审查、环境影响评价、生产许可等制度，把好项目规划、环评和质量关，杜绝引进落后产能。推进园区循环化，加强园区节能减排和资源综合利用，做好园区“三废”污染治理。推进产业生态化，将“生态+”理念融入产业发展全过程，大力发展绿色生态农业，深入实施绿色制造工程，快速发展生态旅游、健康养老等现代服务业，不断提高经济绿色化水平。

　　加强环境治理。统筹推进山水林田湖生态保护和修复，加强大气、水、土壤污染防治，持续削减污染物排放量。全力整改中央环保督察组反馈的突出问题。加大环保“零点行动”力度，开展沿长江、环西海环保专项治理，加强修河流域生态保护。加快推进环庐山绿化提升项目，加强林地和湿地生态保护，完成造林绿化15.2万亩。

　　完善生态文明制度。落实生态保护红线制度，完善生态红线管制措施，加强耕地和基本农田保护。积极探索生态补偿机制，争取更多县（市）列入国家重点生态功能区。严格落实“河长制”。推进环保机构垂直管理体制改革，建立重点企业环保驻厂监察员制度。健全生态文明考核和责任追究制度。培育绿色文化，倡导绿色生活。

　　（九）聚焦生活富裕、精神富有，认真办好民生实事，回应人民群众的新期待。认真落实共享发展理念，持续加大民生投入，不断提高人民群众的幸福感。

　　坚决打好脱贫攻坚战。把脱贫攻坚作为重大政治任务和首要民生工程，以精准扶贫为核心，大力实施“四个一批”脱贫模式，确保10万人脱贫，104个贫困村退出，非贫困县贫困人口、贫困村全部退出。大力实施“一村一品”产业扶贫，进一步增强贫困群众“造血”能力。扎实推进贫困地区基础设施建设，全面改善发展条件。完成扶贫搬迁1万人。继续推进教育脱贫，大力实施“雨露计划”，让更多贫困群众掌握一技之长。深入推进健康扶贫，为贫困户提供基本医疗、大病保险、医疗救助“三重保障”。对完全丧失劳动能力的特困家庭，实行政府兜底脱贫。

　　扎实推进民生工程。创新保障房管理运营模式，继续推行“租售并举”，逐步形成保基本、促公平的公租房保障体系。加强职业技能培训，积极开发公益性就业岗位，加大对“4050”就业困难人员和零就业家庭的帮扶力度，登记就业率稳定在96.5%以上。大力实施“十个一百”惠民利民工程，新建改造绿地小游园、文化健身小广场、公共停车场、标准菜市场、免费公厕、标准化幼儿园、边街小巷、公交候车亭、便民放心超市、社区综合服务站各20个以上。

　　大力发展社会事业。启动实施第三期学前教育行动计划，加大乡镇公办幼儿园建设力度。推动义务教育均衡优质发展，提升特殊教育水平，加快职业教育产教融合，促进高等教育内涵式发展。支持共青科教城三所院校建成招生。大力实施文化惠民工程，力争国家公共文化服务体系示范区创建通过评审。办好纪念周敦颐诞辰1000周年活动。全面推进公立医院综合改革，建立医疗、医保、医药联动机制，健全分级诊疗体系，加强基层卫生服务能力建设，方便群众就医。加强重大传染病防控工作。坚持计划生育基本国策，促进人口均衡发展。推进群众体育、竞技体育和体育产业协调发展。加大食品药品监测力度，保障食品药品安全。推进养老服务体系建设，探索社会化养老新模式。

　　加强和创新社会治理。抓好信访维稳工作，畅通信访渠道，加快诉访分离，压实信访责任，依法及时解决群众合理诉求。完善社会治安防控体系，加强社会治安综合治理，依法严厉打击违法犯罪。完善应急管理机制，增强应急处置能力。深入推进“法治九江”“平安九江”建设，全力维护社会稳定。

　　切实抓好安全生产。严格落实安全生产责任制，建立常态化、规范化的风险管控和预警机制，深入推进隐患排查治理。加大监管执法力度，加强危险化学品、煤矿、非煤矿山、建筑施工、道路交通等重点领域整治，严惩安全生产违法违规行为，确保人民群众生命财产安全。

　　推进军民融合深度发展，加强国防教育、国防动员和后备力量建设，提升双拥工作水平。充分发挥统计、审计、物价、工商、供销、国安、消防、防震减灾、人民防空、盐业、邮政、海事、气象、水文、对台、民族宗教、外事侨务、驻外联络、新闻出版、广播影视、档案、地方志、保密、机构编制、无线电管理、妇女儿童、老龄、残疾人、红十字会、慈善等工作在经济发展和社会建设中的促进作用。

　　（十）聚焦履职担当、廉洁高效，加快转变政府职能，树立实在实干政府的新形象。加快转变政府职能，改进工作作风，提升服务水平，切实提高政府的执行力和公信力。

　　牢固树立法治思维。加快法治政府建设，把政府工作全面纳入法治轨道。强化决策法定程序的刚性约束，健全公众参与、专家论证、风险评估、合法性审查、集体讨论决定制度，确保所有政府行为都于法有据，任何部门都不得法外设权。认真落实民主集中制，主动接受人大的法律监督、政协的民主监督和社会监督。认真办理人大代表议案、建议和政协委员提案，广泛听取民主党派、工商联、老同志和社会各界的意见、建议。

　　保持真抓实干作风。健全抓落实的机制，强化抓落实的责任，量化细化工作任务，做到责任主体明确、工作措施明确、完成时限明确，确保每一项工作有人抓、有人管、有人落实。坚持多到现场去、到工地去、到企业去，面对面帮助企业和群众解决实际困难和问题。严格督促检查，强化跟踪问效，对重点工作和重大事项逐件逐项抓协调、抓督查、抓执行，确保各项工作落地生根。

　　切实强化履职担当。加强调查研究，增强分析问题、解决问题能力，不断提高履职担当的本领。加强公务员队伍建设，完善政绩考核评价体系，健全正向激励机制，带动更多的人干事创业。建立容错纠错机制，旗帜鲜明地为担当者担当、给改革创新者撑腰鼓劲，让想干事的有机会、敢干事的有舞台、会干事的有作为。

　　牢记为民服务宗旨。坚持把群众满意作为政府工作的最终目标和根本追求，时刻牢记全心全意为人民服务的宗旨，把群众的衣食住行、安危冷暖放在心上、抓在手中、落在实处。对于群众普遍关心的养老忧虑、就医苦恼、交通怨言、就学担心、舌尖安全、办事不顺等问题，在充分调研、广泛征求意见的基础上，分轻重缓急，确定一批有针对性的民生实事，认真解决到位。切实兑现向百姓作出的承诺，做不到、做不好的不承诺，承诺的坚决兑现到位。

　　始终把纪律和规矩挺在前面。认真落实党风廉政建设主体责任，认真履行“一岗双责”，更加严格执行中央“八项规定”精神，驰而不息反对“四风”。强化工程建设、政府采购、资金使用等重点领域的监管，做到有腐必反，有贪必肃，有责必问。坚持量力而行、尽力而为，厉行节约，勤俭办事，严控“三公经费”支出。严守法律、道德和良心底线，始终保持清正廉洁本色。

　　各位代表，蓝图鼓舞人心，使命催人奋进。让我们更加紧密地团结在以习近平同志为核心的党中央周围，在省委、省政府和市委的坚强领导下，凝心聚力，真抓实干，奋勇拼搏，为实现九江全面崛起、百姓更加幸福而努力奋斗！(
